# A Physics-Informed Residual and Particle Swarm Optimization Framework for Physics-Informed UAV GPS Spoofing Detection

**DOI:** 10.3390/s25226925

**Published:** 2025-11-13

**Authors:** Ting Ma, Xiaofeng Zhang

**Affiliations:** School of Computer Science, Civil Aviation Flight University of China, Guanghan 618307, China; zhangxiaofeng@cafuc.edu.cn

**Keywords:** UAV, particle swarm optimization, XGBoost, GPS spoofing detection, physics-informed residual

## Abstract

Global Positioning System (GPS) spoofing poses a significant threat to the reliability of unmanned aerial vehicle (UAV) navigation systems that rely heavily on Global Navigation Satellite Systems (GNSS). To address this challenge, we propose a detection framework named PIR–PSO–XGBoost, which integrates Physics-Informed Residual (PIR) modeling with Particle Swarm Optimization (PSO) and Extreme Gradient Boosting (XGBoost). Unlike existing detection frameworks that rely on handcrafted features or deep black box models, the proposed method introduces a physically interpretable residual construction process that captures signal inconsistencies by enforcing temporal and carrier level consistency across GNSS observables. These residuals, combined with conventional navigation features, are used to train an XGBoost-based classifier, while PSO is employed to perform global hyperparameter tuning to enhance model generalization and robustness across diverse spoofing scenarios. This design improves interpretability and computational efficiency, addressing the limitations of traditional feature engineering and deep learning-based detectors. Experimental results on a real-world GPS spoofing dataset demonstrate that the proposed framework achieves a classification accuracy of 95.26% and an F1-score of 95.28%, significantly outperforming conventional learning baselines. These findings confirm that combining physics-guided feature construction with swarm optimized learning yields a robust, efficient, and deployable solution for GPS spoofing detection in UAV applications.

## 1. Introduction

With the rapid advancement of unmanned aerial vehicle (UAV) technology, its application has expanded across numerous critical domains, including traffic monitoring [1,2], disaster surveillance and emergency response [3,4], construction management [5], logistics delivery [6], and search and rescue operations. By leveraging sophisticated autonomous flight control and task execution capabilities, UAVs substantially enhance operational efficiency and safety while reducing reliance on direct human involvement. On one hand, UAVs can operate independently in high-risk or complex environments, thereby mitigating safety hazards commonly associated with manual operations. On the other hand, equipped with intelligent perception and navigation systems, UAVs are able to achieve precise trajectory control while reducing uncertainties arising from human intervention.

Global Positioning System (GPS) spoofing has increasingly emerged as a critical cybersecurity threat to UAV systems. Owing to its stealthy and controllable nature, this attack vector is highly feasible in real-world scenarios. Documented incidents have shown that spoofing is not only technically achievable but also capable of inducing severe mission deviations or even complete system failures, thereby exposing the fragility of UAV navigation security. A notable case occurred in 2011, when a U.S. RQ-170 Sentinel stealth drone was reportedly captured in Iran through a GPS spoofing attack [7]. By emulating authentic satellite signals, the attackers injected falsified positioning data into the UAV navigation module, thereby creating positional errors that diverted the aircraft from its intended trajectory and forced it to land within a controlled zone. This incident revealed that even advanced military UAVs lack sufficient resilience against malicious interference, sparking widespread concerns over the reliability of navigation security mechanisms. Similar risks have been observed in other contexts. In 2017, for example, dozens of vessels in the Black Sea simultaneously experienced navigation anomalies, with some systems misreporting their locations as inland airports situated tens of kilometers away. Although this episode did not directly involve UAVs, it demonstrated the technical feasibility of large-scale GPS spoofing and underscored the vulnerability of autonomous aerial systems operating in open maritime environments. Given UAVs’ frequent use in maritime patrol, payload delivery, and emergency rescue missions, such risks are of particular concern. During the 2022 Russia–Ukraine conflict, Ukrainian reconnaissance UAVs frequently encountered navigation disruptions, including trajectory deviations and communication failures. Battlefield reports suggested that these UAVs, many of which were commercial-grade platforms, either failed to return autonomously or crashed when operating near Russian controlled regions. Analysts attributed these outcomes to electronic warfare systems such as Tobol or Zhitel, which broadcast spoofed Global Navigation Satellite Systems (GNSS) signals to mislead UAV navigation modules, ultimately causing positional errors or complete navigational breakdowns. Collectively, these real-world cases demonstrate that GPS spoofing has evolved from a theoretical concept into a practical and impactful threat. Low-cost UAVs lacking anti-jamming or spoofing detection capabilities are especially vulnerable in contested electromagnetic environments, significantly impairing their reliability and mission effectiveness. Addressing this challenge requires the development of detection and mitigation techniques that are robust, real-time, and generalizable, thereby ensuring secure and stable UAV operations in complex environments.

**Figure 1:**
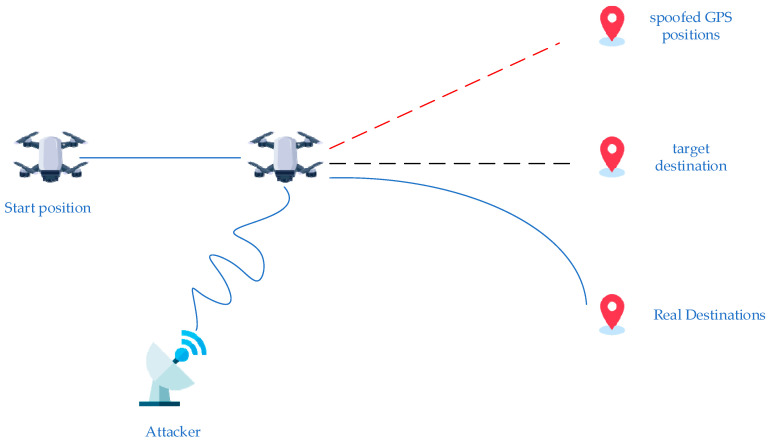
UAV GPS Spoofing Scenario.

GPS spoofing is a type of attack in which falsified or manipulated navigation signals are broadcast to mislead UAVs into receiving erroneous positioning information. Attackers can influence the behavior of target UAVs without triggering system alarms, causing them to deviate from planned flight paths or even enter predefined restricted zones, thereby enabling behavioral interference or potential remote control. Such attacks are highly covert and directly effective, particularly when aimed at commercial or low cost UAV platforms, which often lack sophisticated spoofing countermeasures. Figure 1 presents a representative GPS spoofing attack scenario. In this illustration, a UAV departs from its initial location and navigates toward a designated target destination using standard GNSS signals. A ground based attacker then transmits counterfeit GPS signals with higher power, which override the legitimate satellite transmissions. As a result, the UAV locks onto the spoofed signals and is misled into following a false trajectory toward a spoofed location. This redirection may cause the UAV to enter restricted areas, fail its mission objectives, or even be exposed to hostile interception. The figure highlights the deceptive nature of GPS spoofing and the urgent need for effective detection and mitigation methods in real world UAV applications.

In recent years, a wide range of GPS spoofing detection approaches have been proposed in the academic community, including signal-level feature extraction, spatial consistency verification, multi-source sensor redundancy checks, and machine learning-based intelligent detection techniques. Among these, machine learning methods have attracted considerable attention due to their ability to capture complex nonlinear relationships. Nevertheless, despite notable progress, several critical challenges remain unresolved. First, most models depend primarily on raw observational features, with limited integration of navigation physics and insufficient interpretability. Second, their performance often degrades under varying attack intensities, environmental noise, or dynamic scenarios, resulting in restricted generalization capability. Third, hyperparameter tuning still relies on manual adjustment or static grid search, which hinders adaptability and reduces real-time responsiveness and deployment flexibility.

To address these challenges, this study proposes a GPS spoofing detection framework that integrates Physics-Informed Residual modeling with a Particle Swarm Optimization (PSO) strategy. By exploiting intrinsic physical correlations among GNSS observables, the method constructs a set of residual indicators that capture spoofing-induced anomalies, including variations in pseudorange errors, carrier phase offsets, and fluctuations in signal-to-noise ratios. Based on these features, a continuous space PSO algorithm is employed to globally optimize the hyperparameters of an XGBoost-based classifier, thereby improving detection accuracy and generalization across diverse spoofing scenarios. Experimental evaluations on a real-world GNSS spoofing dataset demonstrate that the proposed PIR–PSO–XGBoost framework achieves a classification accuracy of 95.26% in multiclass detection tasks, significantly outperforming models that rely solely on raw navigation features. In addition to delivering high detection performance, the framework offers enhanced interpretability, efficient parameter tuning, and fast convergence, demonstrating its practicality and scalability for real-world UAV applications. The main contributions of this study are summarized as follows:

- A novel Physics-Informed residual feature modeling mechanism is introduced: Inspired by the concept of Physics-Informed Neural Networks, the method constructs multidimensional residual signals from the interdependencies among GNSS navigation observables, effectively capturing spoofing-induced physical anomalies and providing the detection model with high-level features that are both interpretable and time-sensitive.
- A PSO-based hyperparameter optimization strategy is proposed: To overcome performance bottlenecks in Extreme Gradient Boosting (XGBoost) parameter tuning, a continuous space PSO algorithm is employed to globally and jointly optimize key hyperparameters (such as maximum tree depth, learning rate, regularization coefficients, and sampling rate), thereby enhancing model robustness and generalization in complex spoofing scenarios.
- The proposed model is empirically validated using a real-world GNSS spoofing dataset: Experimental results demonstrate that the proposed PIR–PSO–XGBoost framework achieves a classification accuracy of 95.26% in multiclass GPS spoofing detection tasks, substantially outperforming conventional models that rely solely on raw navigation features. These findings confirm the practical utility of the framework and underscore its performance advantages in real-world UAV scenarios.

The structure of this paper is organized as follows: Section 2 provides a review of related work, focusing on recent advancements in GPS spoofing detection within UAV systems. Section 3 outlines the architecture of the proposed detection framework, which integrates Physics-Informed Residual modeling, Particle Swarm Optimization, and the XGBoost classifier. Section 4 details the experimental design, including dataset sources, the construction of spoofing scenarios, feature extraction methods, and evaluation metrics. Section 5 presents the experimental results and performance analysis, including comparisons with baseline models and ablation studies. Section 6 examines the advantages and limitations of the proposed method, alongside potential avenues for future improvement. Finally, Section 7 concludes the study, emphasizing the practical significance and application prospects of the proposed approach in enhancing UAV navigation security.

## 2. Related Works

As UAVs continue to evolve, numerous detection strategies have been proposed to counter GPS spoofing attacks. Feng et al. [8] proposed a two-stage training framework for detecting UAV hijacking attempts, integrating a genetic algorithm (GA) to optimize training parameters and XGBoost to enhance prediction accuracy and model stability, forming a GA–XGBoost hybrid model. Real-time validation was conducted using a commercial multicore embedded platform and autopilot sensor board, yielding a detection accuracy of 96.3% for non-hijacked UAVs, with faster response times than mainstream approaches. Sun et al. [9] developed a deep learning model based on a PCA–CNN–LSTM architecture to detect GPS spoofing signals in small UAVs. The framework included signal preprocessing, dimensionality reduction, and feature extraction via principal component analysis (PCA), local pattern extraction using convolutional neural networks (CNN), and temporal modeling with long short-term memory (LSTM) networks. Experimental results demonstrated up to 99.49% detection accuracy, showing strong performance in complex nonlinear navigation scenarios. Talaei et al. [10] proposed two dynamic model selection strategies—Metric Optimized Dynamic Selector (MODS) and Weighted MODS—to detect cyberattacks in UAV systems. These strategies integrated ten distinct machine learning models to improve adaptability and detection performance, with simulation-based evaluations indicating a peak accuracy of 99.6%. Wu et al. [11] introduced a CNN–BiLSTM–Attention model that used attention mechanisms to enhance the representation of critical features, effectively identifying denial of service (DoS) and GPS spoofing attacks with 99.1% accuracy, validating its strength in temporal modeling and complex attack scenarios. Alhoraibi et al. [12] developed a GPS spoofing detection framework based on adversarial machine learning (AML), employing generative adversarial networks (GANs) to produce adversarial samples, which were then processed through CNN and LSTM components in a dual-stage structure. This framework achieved excellent performance on a large-scale dataset, reaching up to 98% accuracy. Alanazi et al. [13] proposed a GPS spoofing detection framework combining self-supervised representation learning (SSRL) with transfer learning. It integrated LSTM–GRU, LSTM–RNN, and deep neural network (DNN) architectures to enable automatic feature extraction and improve generalization under limited labeled data. The hybrid SSRL–transfer learning model significantly improved spoofing detection capability in real-world UAV applications. Al Syouf et al. [14] presented a framework that incorporated multiple machine learning models with a hybrid recursive feature elimination method (GBM–RFE), combining Spearman correlation analysis with gradient boosting machines to construct an optimal feature subset. Their Bagging model achieved 99.50% accuracy with a 0.029 s delay on the GPS spoofing dataset, and 99.16% accuracy with only 0.002 s inference time on the UAV position spoofing dataset. Al Sabbagh et al. [15] proposed a hybrid deep learning approach combining GA and LSTM to enhance UAV resilience against GPS spoofing. By expanding the GA search space to optimize LSTM hyperparameters—including the number of layers, units, and dropout rates—they improved detection accuracy from 88.42% to 93.12%, along with a significant boost in F1-score. Xue et al. [16] introduced a detection framework based on satellite image matching, comparing real-time aerial imagery with historical satellite images aligned to GPS coordinates. Although highly accurate in static environments, this approach required significant image storage and interruptions for ground image capture, limiting real-time applicability. Barak et al. [17] developed a video-stream-based detection mechanism that correlated inter-frame similarity with GPS coordinate distances. By fitting a similarity–distance curve, they detected anomalous GPS behavior without relying on stored imagery. However, its performance degraded in complex terrain, variable lighting, or fluctuating altitude. Elena et al. [18] proposed a probabilistic detection approach using Kullback–Leibler (KL) divergence. UAV parameters such as visible satellite count, speed, altitude, and geographic coordinates were modeled using Poisson distributions, and anomalies were detected by measuring divergence from expected values. This method performed well in simulations but was sensitive to benign anomalies caused by natural variations or sudden flight state transitions, potentially triggering false alarms. A study by Mouzai et al. [19] explored the use of multi-channel GPS receivers in detecting spoofing attacks, applying tree-based machine learning models such as decision trees, random forests, and XGBoost to classify GPS signals and effectively detect spoofing.

In addition to conventional machine learning and deep learning-based frameworks, recent research has introduced adaptive and intelligent methodologies to enhance the detection and mitigation of GPS spoofing threats. Hu et al. [20] proposed a reinforcement learning-driven framework that integrates detection and mitigation mechanisms in a unified structure, enabling unmanned aerial vehicles to dynamically respond to spoofing attacks based on environmental feedback and learned policies. This approach emphasizes the potential of autonomous defense strategies that go beyond static classification boundaries. Complementing this line of work, Abrar et al. [21] developed GPS-IDS, an anomaly-based intrusion detection system designed for autonomous vehicles. Their method leverages statistical and physical inconsistencies in GNSS observations to detect spoofing behavior without requiring extensive labeled data. These studies collectively demonstrate a shift toward more adaptive, context-aware spoofing detection models capable of maintaining performance across diverse and evolving threat conditions.

Table 1 presents a comparative summary of representative GPS spoofing detection methods. As shown, most deep learning models lack interpretability and require significant computational resources. PINN-based approaches embed physical knowledge implicitly, limiting transparency and flexibility. In contrast, the proposed PIR–PSO–XGBoost framework explicitly constructs interpretable residual features from GNSS physics and achieves high accuracy with low computational cost, addressing key limitations of prior methods.

Although previous studies have explored GPS spoofing detection through statistical modeling, deep learning, and physics-informed approaches, existing methods often involve trade-offs among interpretability, adaptability, and computational cost. Deep learning architectures, despite their high accuracy, typically lack transparency and impose substantial inference overhead, limiting their applicability on real-time and resource-constrained UAV platforms. Physics-inspired frameworks, such as those based on PINNs, embed domain knowledge within neural networks implicitly, which may obscure feature-level insight and complicate parameter tuning. In contrast, the proposed PIR–PSO–XGBoost framework constructs explicit Physics-Informed Residual features that directly reflect GNSS signal inconsistencies, thereby enhancing model transparency and supporting lightweight implementation. These residuals, together with conventional navigation parameters, are used to train an XGBoost-based classifier whose hyperparameters are globally optimized using Particle Swarm Optimization. This strategy improves detection accuracy and robustness across diverse spoofing types, while avoiding the limitations of manually fixed parameters and exhaustive grid search procedures. By integrating physically grounded feature design with swarm-optimized learning, the proposed framework bridges the gap between explainability and performance, providing a deployable and efficient solution for real-time UAV navigation security.

Recent advancements in hybrid physics-informed regression methods, particularly in the reliability modeling domain, have shown great promise in improving predictive accuracy and robustness under uncertainty. For instance, the state-of-the-art physics-embedding framework introduced by [22] integrates physical constraints directly within regression models, improving the model’s ability to adapt to noisy and non-stationary data. In contrast, the PIR–PSO–XGBoost framework presented here differs in that it explicitly constructs residual features reflecting GNSS signal inconsistencies, rather than embedding physical constraints implicitly within the model. This explicit residual design enhances interpretability and reduces computational overhead, making it highly suitable for real-time applications in resource-constrained UAV platforms. The connection between our work and recent developments in physics-embedding methods highlights its cross-domain applicability, showcasing its potential use in other fields such as reliability engineering, where robustness against adversarial conditions is crucial.

## 3. Proposed Method

This study proposes a GPS spoofing detection approach that integrates physics-informed residual modeling with a PSO strategy. The proposed approach combines a physics-consistent feature construction mechanism with intelligent hyperparameter optimization and leverages an XGBoost classifier for efficient classification. It aims to enhance the recognition accuracy and detection robustness of UAV systems when operating under complex environmental conditions.

### 3.1. System Framework Overview

**Figure 2:**
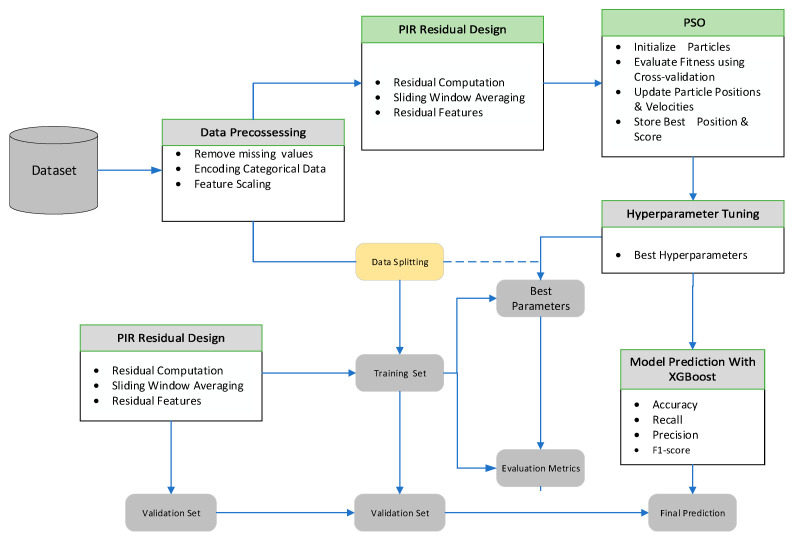
Overall architecture of the proposed GPS spoofing detection framework integrating Physics-Informed Residual feature construction and Particle Swarm Optimization-based hyperparameter tuning for an XGBoost-based classifier.

Figure 2 depicts the overall workflow of the proposed PIR–PSO–XGBoost framework, which comprises four key modules: data preprocessing, residual feature construction based on physics-informed principles, hyperparameter optimization using particle swarm optimization, and classification modeling with XGBoost. The preprocessing module addresses missing or redundant values and standardizes input variables to ensure data consistency and model readiness. The standardized data is then fed into the residual construction module, which extracts time-series features by enforcing physical relationships among GNSS observables through a sliding window mechanism. These residuals, such as carrier phase differences, pseudorange deviations, and abrupt fluctuations in signal-to-noise ratio, enhance the model’s sensitivity to spoofing-induced anomalies while improving interpretability. The enriched feature set is subsequently divided into training and testing subsets. PSO is used to perform global hyperparameter optimization of the XGBoost classifier, guided by five-fold cross-validation and an early stopping mechanism. Finally, the optimized XGBoost model classifies normal and spoofed signals. By combining physically consistent residual modeling with swarm-based optimization, the framework achieves high accuracy, robustness, and generalization across complex and dynamic GPS spoofing scenarios, thereby supporting its applicability for real-time UAV navigation security.

The pseudocode of the proposed algorithm is presented in Algorithm 1.
**Algorithm 1.** PIR–PSO–XGBoost Hyperparameter Optimization**Require:** Raw GPS dataset D, PSO population size P, maximum iterations T**Ensure:** Optimized XGBoost model $M^{*}$    1:  **Data Preprocessing**    2: $D_{\mathrm{clean}}$← RemoveMissingValues(D)    3: $X_{base},y$←ExtractBaseFeatures($D_{\mathrm{clean}}$)    4: $X_{pinn}$←Compute PIR Residuals($D_{\mathrm{clean}}$)    5: $X_{all}$←Concatenate($X_{base},X_{pinn}$)    6: $X_{scaled}$←Standardize($X_{all}$)    7:  $X_{train},X_{test},y_{train},y_{test}$←StratifiedSplit($X_{scaled},y$)    8: **Initialize PSO Swarm**    9:  Swarm←InitializeParticles(P,bounds)    10: $g_{best\_pos},g_{best\_score}$←None,∞
    11:  **PSO Optimization Loop**    12: **for** t = 1 **to** T **do**    13:   Update inertia weight w←$w_{max}$ − $\frac{t}{T}(w_{max}-w_{min})$
    14:   **for** each particle p∈Swarm **do**    15:    $score_{p}$←1-CVAccuracy($X_{train},X_{test},P$.position)    16:    **if** ${\mathrm{score}}_{p}$ < P.best_score **then**    17:       Update personal best p.best_position←P.position    18:       Update personal score p.best_score←${\mathrm{score}}_{p}$
    19:    **if** ${\mathrm{score}}_{p}$ < $g_{best\_score}$ **then**    20:       Update global best $g_{best\_pos}$←P.position    21:       Update global score $g_{best\_score}$←$score_{p}$
    22:   **end for**    23:   **for** each dimension j of p**do**    24:     Update velocity: $v_{j}$←$w\times v_{j}+\gamma_{1}(p_{best,j}-p_{j})+\gamma_{2}(g_{best,j}-p_{j})$ // γ_1_: individual factor, γ_2_: social factor    25:     Clamp and update position within bounds    26:   **end for**    27: **end for**    28: Model Training and Evaluation    29: $M^{*}$←TrainXGBoost($X_{train},y_{train},g_{best\_pos}$)    30: $\hat{y}$←$M^{*}.Predict(X_{test})$
    31: Evaluate Accuracy, Precision, Recall, F1-score    32: **Return:** Optimized model $M^{*}$


### 3.2. Physics-Informed Residual Feature Design

This study leverages the principles of Physics-Informed Neural Networks to construct a set of residual features that capture the inherent physical relationships among GNSS navigation observables. These features are specifically designed to characterize anomalous signal behavior and enhance the interpretability of data-driven detection models. In practical navigation systems, many observed variables adhere to deterministic or approximately linear physical constraints. For instance, the difference between carrier phase cycles (CP) and pseudorange (PD) is expected to remain stable under normal conditions, while pseudorange values across channels, such as PD and the Prompt Quadrature Component, should exhibit high consistency. Additionally, variables like carrier-to-noise ratio (C/N0), time of week (TOW), and correlator outputs typically show continuous and physically interpretable trends that reflect the underlying system dynamics. GPS spoofing attacks often disrupt these physical regularities, leading to noticeable discrepancies among correlated variables. These deviations serve as crucial indicators for spoofing detection. Based on this insight, the proposed framework extracts nine categories of Physics-Informed Residual features from raw GNSS data, as summarized in Table 2.

To improve the mathematical clarity and physical interpretability of the proposed residual features, we present formal expressions for three representative indicators constructed from GNSS observables. These residuals are intended to uncover inconsistencies in signal propagation, correlator response, and power variation induced by spoofing activities:

(1) $$\mathrm{res}\_\mathrm{TCD}\_\mathrm{jump}=\left|TCD^{\left(t\right)}-TCD^{\left(t-1\right)}\right|$$

(2) $$\mathrm{res}\_\mathrm{RX}\_\mathrm{var}=\mathrm{Var}(RX^{\left(t\text{-}\mathrm{window}\right)},RX^{\left(t\text{-}\mathrm{window}+1\right)},\cdots ,RX^{\left(t\right)})$$

(3) $$\mathrm{res}\_\mathrm{CN}0\_\mathrm{slope}=CN0^{\left(t\right)}-CN0^{\left(t-w\right)}$$

where t denotes the current observation epoch, and ω is the length of the sliding window used for residual calculation. t represents the current observation epoch, which is the specific time of the measurement. ω refers to the length of the sliding window, determining how many previous data points are considered in the residual calculation. The term window is the size of the sliding window used in res_RX_var and res_CN0_slope, indicating the number of consecutive data points included for variance and slope calculations.

**Figure 3:**
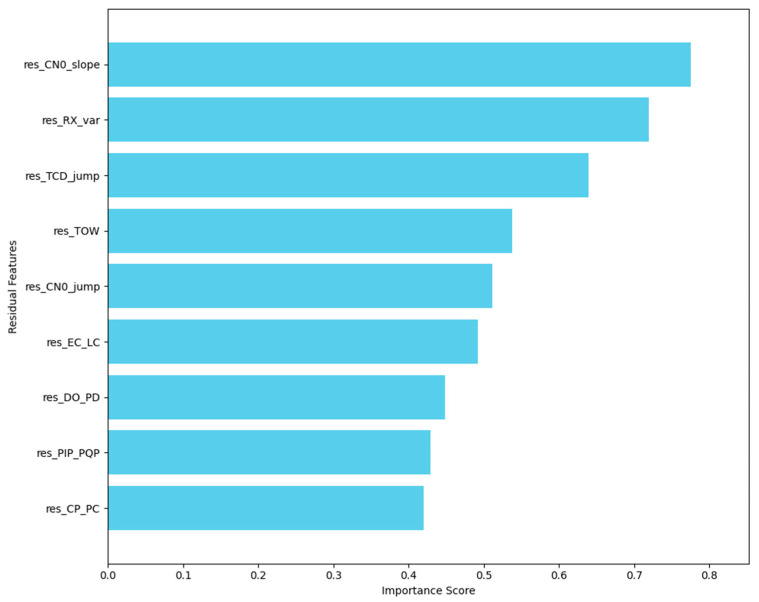
Feature Importance Analysis of Residuals.

Figure 3 presents the feature importance analysis, with res_CN0_slope, res_RX_var, and res_TCD_jump ranked as the most influential features for detecting GPS spoofing. These features exhibit higher importance scores, highlighting their critical role in distinguishing between authentic and spoofed GPS signals. The elevated importance of these features is likely attributed to their sensitivity to signal timing and satellite-receiver distance inconsistencies, both of which are key factors in spoofing detection scenarios.

As shown in the figure, other significant features, such as res_TOW, res_CN0_jump, and res_EC_LC, provide additional temporal and signal quality information, further aiding the model in detecting subtle variations indicative of spoofing. The distribution of importance scores reveals that no single feature dominates the model’s decision-making process. Instead, it is the combination of these residual features that enables the model to effectively capture and identify complex spoofing attacks.

All residual features are normalized and concatenated with the original navigation features to form the model input, thereby enhancing the model’s sensitivity to physical anomalies. This residual construction strategy not only improves the discriminative power of the features but also contributes to better model interpretability and robustness, providing physics-informed support for subsequent classification modeling.

### 3.3. PSO

The PSO algorithm is a population-based metaheuristic inspired by swarm intelligence, simulating the information-sharing behavior observed in bird flocks during foraging. It conducts a collaborative search within the solution space using a swarm of particles, where each particle represents a candidate solution corresponding to a specific combination of hyperparameters. The position of a particle indicates its current solution, while its velocity determines the direction and magnitude of its movement. Particles are influenced by both their personal best positions (individual experience) and the global best position identified by the swarm (social experience). The main steps of the PSO algorithm are as follows:

- Initialization: A set of particles is randomly initialized within the solution space, with each particle defined by a position and a velocity vector. The position encodes a candidate solution (e.g., a set of model hyperparameters), while the velocity determines the direction and step size of movement within the search space. Key parameters, such as swarm size, maximum number of iterations, inertia weight, cognitive coefficient, and social coefficient, are configured to control the update dynamics of particles and balance global exploration with local exploitation, guiding the swarm to progressively converge toward the global optimum.
- Fitness Evaluation: The fitness of each particle is evaluated at its current position. In this study, the fitness function is defined as the average classification accuracy obtained through K-fold cross-validation. The fitness evaluation formula is given as follows:
  (4) $$F\mathrm{itness}=1-MeanACC_{cy}$$
  Here, $\mathrm{Mean}ACC_{cy}$ denotes the average accuracy obtained from K-fold cross-validation. A lower fitness value indicates that the corresponding hyperparameter combination yields better performance
- Individual Best Update: For each particle, if the current fitness value surpasses its personal best fitness value, the personal best position and the corresponding fitness value are updated.
- Global Best Update: Among the personal best positions of all particles, the one with the highest fitness value is selected as the current global best position. This position serves as a crucial reference point, guiding the swarm’s overall search direction and steering the entire population toward the global optimum.
- Velocity and Position Update: The velocity and position of each particle are updated according to the following equations:
  (5) $$v_{i}^{\left(t+1\right)}=w\cdot v_{i}^{\left(t\right)}+c_{1}\cdot r_{1}\cdot (p_{i}^{best}-x_{i}^{\left(t\right)})+c_{2}\cdot r_{2}\cdot (g^{best}-x_{i}^{\left(t\right)})$$
  (6) $$x_{i}^{\left(t+1\right)}=x_{i}^{\left(t\right)}+v_{i}^{\left(t+1\right)}$$
  In the update equations, $x_{i}^{\left(t+1\right)}$ represents the position of particle i at iteration t+1, and $v_{i}^{\left(t+1\right)}$ denotes its velocity at the same iteration. The parameter w is the inertia weight, which controls the balance between exploration and exploitation during the search process. The coefficients $c_{1}$ and $c_{2}$ are the cognitive and social learning factors, respectively.$r_{1}$ and $r_{2}$ are random values independently drawn from a uniform distribution in the range [0, 1]. The variable $p_{i}^{best}$ denotes the best historical position found by particle i, and $g^{best}$ refers to the global best position currently identified by the swarm.
  If any dimension of a particle’s updated position exceeds the predefined boundary of the search space, a boundary handling strategy is applied to maintain feasibility. In practice, the out-of-range value can either be clipped to the nearest boundary value or reflected back into the valid region. This mechanism ensures that all particles remain within the search domain, preventing divergence during the optimization process.
- Termination Criteria: The algorithm terminates when the maximum number of iterations is reached or when the global best fitness value shows no improvement over a specified number of consecutive generations, indicating convergence. At this point, the global best solution identified during the search process is returned as the final output.

In this study, Particle Swarm Optimization is used to explore a seven-dimensional hyperparameter space, which includes maximum tree depth, learning rate, subsample ratio, feature sampling ratio, regularization coefficient, minimum loss reduction, and the number of boosting rounds. The search ranges for these hyperparameters, as shown in Table 3, are optimized within predefined limits to ensure robust model performance across various spoofing scenarios. The fitness of each particle is evaluated using five-fold cross-validation, with model performance assessed on distinct data subsets to verify its generalizability. To minimize the risk of overfitting, an EarlyStopping strategy is incorporated to terminate the training process when no further improvements are observed, ensuring the model’s ability to generalize to unseen data.

### 3.4. XGBoost

XGBoost is an ensemble learning method built upon the gradient boosting framework. It is known for its high computational efficiency, strong predictive power, and excellent scalability. The algorithm constructs decision trees incrementally, with each successive tree trained to capture the residual errors of the previous iteration, thereby improving overall predictive accuracy. Compared to conventional Gradient Boosted Decision Trees, XGBoost introduces enhancements in model representation, regularization mechanisms, and optimization techniques, making it particularly effective for classification and regression tasks involving structured datasets.

The objective function of XGBoost is defined as follows:

(7) $$L=\sum_{i=1}^{n}l(y_{i},{{\hat{y}}_{i}}^{\left(t\right)})+\sum_{k=1}^{t}\Omega (f_{k})$$

(8) $$\Omega (f)=\gamma T+\frac{1}{2}\lambda \sum_{j=1}^{T}w_{j}^{2}$$

Here, L denotes the overall objective function. The first term represents the prediction error, and the second term $\Omega (f_{k})$ is a regularization component that penalizes the complexity of each individual tree $f_{k}$ Specifically, n denotes the total number of training samples, $y_{i}$ denotes the ground truth label of the *i*-th instance, and ${\hat{y}}_{i}^{\left(t\right)}$ denotes the prediction made by the model at iteration t. $f_{k}$ represents the prediction function of the *k*-th regression tree. T denotes the number of leaf nodes in the tree, and $w_{j}$ is the output value of the *j*-th leaf node. γ is the penalty parameter that controls the number of leaf nodes. A larger value of γ encourages the generation of smaller trees. λ is the L2 regularization coefficient for the leaf weights $w_{j}$, which helps to shrink their magnitudes when increased.

## 4. Experimental Setup

All experiments were conducted on a laptop equipped with an AMD Ryzen 7 7840HS processor, 16 GB of DDR5 RAM, and an NVIDIA GeForce RTX 5060 Laptop GPU (NVIDIA Corporation, Santa Clara, CA, USA). The framework was implemented in Python 3.10.9 and executed within the PyCharm 2024.2.6 integrated development environment on the Windows 11 operating system. Model training, feature extraction, and evaluation were performed using standard libraries, including NumPy2.0.2, Pandas2.3.1, SciPy1.13.1, Scikit-learn1.6.1, Matplotlib3.9.4, and XGBoost. GPU acceleration was employed during the training phase to enhance computational efficiency. However, the inference process and residual feature computation primarily rely on lightweight arithmetic operations and decision tree traversal, which can be efficiently executed on general-purpose processors without GPU support. This characteristic suggests that the proposed method can be adapted for deployment on resource-constrained platforms, such as embedded UAV navigation modules or edge computing devices.

### 4.1. Dataset and Preprocessing

The dataset [23] used in this study was developed through a combination of empirical data collection and controlled simulation of GPS spoofing scenarios. Authentic GNSS signals were collected using an eight-channel GPS receiver installed on a vehicle operating at speeds ranging from zero to sixty miles per hour, simulating the dynamic motion characteristics of unmanned aerial vehicles. Additional data were gathered from static positions on rooftops at varying elevations to replicate hovering conditions. Each sample contains thirteen navigation-related features extracted from different stages of the GNSS signal processing chain, including acquisition, tracking, and correlation modules.

Spoofed signals were generated using a software-defined radio (SDR) platform, configured to replicate three typical categories of spoofing attacks. The first category, called simplistic spoofing, involved the transmission of high-power counterfeit signals without synchronization to authentic satellite broadcasts, leading to abrupt deviations in pseudorange and Doppler frequency measurements. In the second category, intermediate spoofing, the simulated signals were synchronized with authentic ones in both code phase and carrier frequency, enabling a smooth and covert displacement of the navigation solution. The most advanced category, sophisticated spoofing, emulated a full satellite constellation using synchronized multi-channel transmissions, allowing comprehensive control over signal parameters and inducing highly realistic positional errors. All spoofing configurations were experimentally verified with a commercial GNSS receiver to ensure signal acquisition, tracking stability, and effective navigation manipulation.

This dataset offers a structured and reproducible foundation for evaluating spoofing detection methods under varying levels of adversarial complexity. It consists of approximately 510,000 samples, with 55 percent corresponding to authentic GPS signals and 45 percent to spoofed signals generated through the three attack categories: simplistic spoofing, intermediate spoofing, and sophisticated spoofing.

- Simplistic Spoofing: In this attack type, the adversary lacks precise knowledge of the receiver’s position and attempts to disrupt the system by transmitting high-power counterfeit GPS signals that are not synchronized with authentic transmissions. These signals typically cause significant pseudorange deviations and abnormal Doppler frequency shifts exceeding 20 Hz, potentially destabilizing the navigation solution. Due to their abrupt and easily recognizable characteristics, spoofed signals in this scenario can be effectively detected using conventional methods that identify anomalies in amplitude or frequency. Although relatively simple to execute, simplistic spoofing is less covert and more easily detectable.
- Intermediate Spoofing: In this attack, the adversary has knowledge of the receiver’s position and generates counterfeit signals that remain closely aligned with authentic ones in both code phase and carrier frequency. This alignment allows for gradual disruption and eventual takeover of the receiver’s navigation solution. Compared to simplistic spoofing, intermediate spoofing is much harder to detect using conventional methods, as the counterfeit signals closely resemble genuine transmissions. However, subtle indicators, such as irregularities in carrier phase behavior, abnormal variations in correlator output amplitudes, and discontinuities in time-of-week information, can provide valuable evidence for its detection.
- Sophisticated Spoofing: This advanced attack utilizes multi-antenna synchronization or software-defined radio platforms to simulate a complete GPS satellite constellation. The attacker can disrupt multiple receiver channels simultaneously, gaining full control over the positioning system. Sophisticated spoofing achieves a high level of realism in both spatial structure and signal characteristics, making it extremely difficult to detect using traditional correlator-based or rule-based algorithms. It is considered one of the most severe threats to navigation system security.

Table 4 presents the output labels and the corresponding sample size distribution for the four categories of GPS signals, including normal signals and the three types of spoofing attacks.

Table 5 summarizes the UAV related parameters included in the dataset:

These parameters collectively describe the GPS signal profile from both spatial and temporal perspectives. The feature set incorporates physical propagation characteristics and internal signal processing responses, forming a multiscale, cross-domain representation framework. This diversity is crucial for capturing anomalies in signal structure, temporal synchronization, and dynamic behavior induced by spoofing attacks. Furthermore, this representation serves as a solid foundation for downstream tasks, including feature importance analysis, residual-based physical modeling, and performance evaluation, ultimately enabling effective detection and precise differentiation among spoofing attack types.

To ensure the reliability and stability of the input data, a preprocessing procedure was applied. Samples containing missing or invalid observations were removed to prevent noise in feature construction and model training. Thirteen fundamental attributes directly associated with GPS navigation signals were retained as the initial input feature set. To enhance the model’s sensitivity to physical inconsistencies, an additional residual feature construction module was introduced based on the principle of physical consistency. Inspired by the Physics-Informed Neural Network framework, this module employs a sliding window mechanism and enforces constraints among correlated variables to extract nine categories of Physics-Informed Residual features. These residuals capture subtle perturbations and dynamic irregularities in GNSS signals under spoofing conditions, improving both the discriminative power and interpretability of the feature representation. After feature construction, all variables were standardized to eliminate scale disparities across dimensions and improve training stability. Finally, the dataset was partitioned into training and testing subsets using a stratified sampling strategy, ensuring class balance and providing a robust foundation for subsequent model development and evaluation.

All performance metrics reported in the Results section, including accuracy, precision, recall, and F1-score, were calculated exclusively based on the independent test set. The dataset was split using stratified sampling, with 70 percent allocated for training and 30 percent reserved for testing. The test set remained entirely isolated throughout all stages of model development, including feature construction, parameter tuning, and model training. This separation ensures that the evaluation results accurately reflect the true generalization performance of the proposed method.

### 4.2. Evaluation Metrics

To comprehensively evaluate the model’s performance in GPS spoofing signal classification, several evaluation metrics derived from the confusion matrix were used. Specifically, these metrics include Accuracy, Precision, Recall, and F1-score, which together provide a multifaceted assessment of the classifier’s detection performance. Accuracy measures the overall correctness of the model’s predictions across all samples and is defined as follows:

(9) $$\mathrm{Accuracy}=\frac{TP+TN}{TP+TN+FP+FN}$$

Precision indicates the proportion of true positive cases among all instances that the model predicts as spoofed signals. It is calculated as follows:

(10) $$\mathrm{Precision}=\frac{TP}{TP+FP}$$

Recall represents the proportion of actual positive instances identified by the model as spoofed signals. It is defined as follows:

(11) $$\mathrm{Recall}=\frac{TP}{TP+FN}$$

The F1-score is defined as the harmonic mean of Precision and Recall. It provides a balanced evaluation of the model’s performance, particularly under conditions of class imbalance. It is calculated as follows:

(12) $$F1\text{-}\mathrm{score}\;=\;2\times \frac{\mathrm{Precision}\times \mathrm{Recall}}{\mathrm{Precision}\;+\;\mathrm{Recall}}$$

In these formulas, *TP* (True Positive) represents the number of spoofed signals correctly identified by the model; *FP* (False Positive) refers to the number of normal signals incorrectly classified as spoofed signals; *FN* (False Negative) indicates the number of spoofed signals that were not detected; and *TN* (True Negative) denotes the number of normal signals correctly classified by the model.

To minimize random errors caused by sample partitioning and enhance the reliability of model evaluation, a five-fold stratified cross-validation strategy was incorporated into the fitness function of the Particle Swarm Optimization algorithm. In this procedure, the training dataset is divided into five subsets, ensuring that the class distribution in each subset is consistent with that of the full dataset. During each fold, four subsets are used for training the model, and the remaining subset is used for validation. After completing five iterations, the average validation performance is calculated and used as the fitness score for the given hyperparameter configuration. This approach significantly improves the model’s generalization ability and reduces the likelihood of overfitting. Additionally, to further enhance training stability and computational efficiency, an Early Stopping mechanism is applied during each training cycle. When validation performance fails to improve for a predefined number of consecutive epochs, the training process is terminated early. This reduces unnecessary computations, accelerates convergence, lowers the risk of overfitting, and strengthens the robustness of the optimization process.

### 4.3. Model Evaluation and Overfitting Prevention

Although the model achieves high accuracy, it is crucial to consider the risk of overfitting, particularly given the optimization of seven hyperparameters using Particle Swarm Optimization. To address this concern, 5-fold cross-validation was employed to assess the model’s generalization capability. This technique ensures that the model’s performance is not overly dependent on a single data split, providing a more reliable estimate of its ability to generalize to new data. The results from cross-validation indicate that the model’s performance remained stable across the five folds, with accuracy values ranging from 0.9494 to 0.9506. This narrow range suggests consistent performance, minimizing the risk of overfitting to the training data.

**Figure 4:**
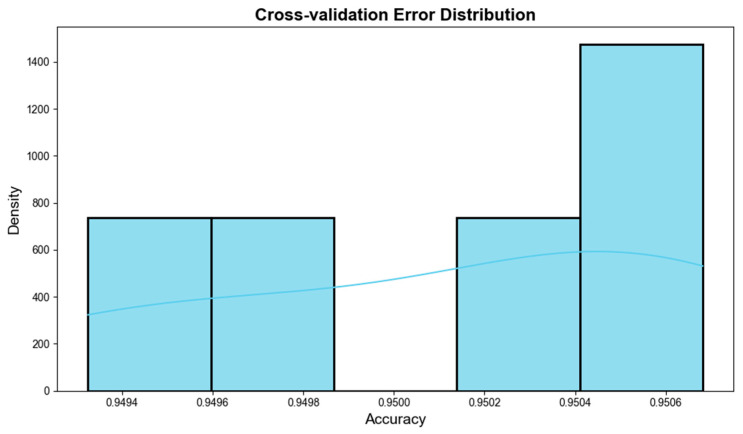
Accuracy Distribution Across Cross-Validation Folds.

Figure 4 displays the accuracy distribution across the five folds. The accuracy values are tightly clustered, and the kernel density estimate (KDE) curve shows a smooth distribution, further confirming the model’s consistency across different data subsets. Additionally, the comparison between the training and test set performances showed a minimal difference (less than 1%), suggesting that overfitting is not a concern. A significant gap between these performances would typically indicate overfitting, but this was not observed.

To further mitigate the risk of overfitting, regularization techniques were applied to control the complexity of the XGBoost model. Additionally, early stopping was employed to halt the training process when the model’s performance on the validation set no longer improved, thus preventing it from fitting noise in the training data.

In summary, through cross-validation, regularization, and early stopping, we ensured that the model’s high accuracy reflects its strong generalization ability rather than overfitting.

## 5. Results and Analysis

### 5.1. Overall Detection Performance

To evaluate the classification performance of different approaches for GPS spoofing detection, seven baseline models were selected, including traditional machine learning algorithms such as Logistic Regression and K-Nearest Neighbors (KNN), the ensemble method XGBoost, and several widely used deep learning architectures: Convolutional Neural Network Recurrent Neural Network (RNN), Long Short-Term Memory, and Multilayer Perceptron (MLP). The proposed PIR–PSO–XGBoost framework was also integrated into the same experimental setup to enable a comprehensive and fair comparison. All models were trained under identical conditions using the same feature set and consistent train–test partitioning to ensure the fairness and reproducibility of the evaluation. The comparative performance results are summarized in Table 6.

As shown in Table 6, traditional shallow models such as Logistic Regression (LR) and K-Nearest Neighbors exhibit limited effectiveness when applied to high-dimensional temporal features. For instance, the LR model achieved an F1-score of only 68.25%, reflecting its inability to capture complex spoofing patterns. In contrast, deep learning models, including LSTM, RNN, CNN, and MLP, demonstrated significantly stronger capabilities in feature extraction and temporal sequence modeling, with all architectures achieving detection accuracies near or above 90%. Among these, the MLP model delivered the best performance, with an accuracy of 91.49% and an F1-score of 91.51%. The XGBoost model, which employs gradient boosting, also yielded competitive results without relying on deep network structures, attaining an accuracy of 89.86%, surpassing most conventional machine learning methods.

The proposed PIR–PSO–XGBoost framework achieved the highest performance across all evaluation metrics, with an accuracy of 95.26%, an F1-score of 95.28%, precision of 95.34%, and recall of 95.26%. These improvements can be attributed to the incorporation of Physics-Informed Residual features that capture spoofing-induced signal variations, along with a Particle Swarm Optimization strategy that enhances the adaptability and generalization capability of the classifier. Overall, PIR–PSO–XGBoost consistently outperformed all benchmark models, demonstrating the practical advantages of combining physics-based feature construction with intelligent optimization for GPS spoofing detection.

### 5.2. Ablation Study

To systematically assess the individual and combined contributions of the key components within the proposed PIR–PSO–XGBoost framework for GPS spoofing detection, a series of targeted ablation experiments were conducted. These experiments focused on two critical modules: physics-consistent modeling, represented by Physics-Informed Residual features, and hyperparameter optimization via Particle Swarm Optimization. Four distinct model configurations were established to facilitate comparative analysis, with the aim of quantifying the performance impact of each module on the overall detection capability. The specific configurations are outlined as follows:

- XGBoost: This baseline configuration uses only the original navigation features, without incorporating residual modeling or hyperparameter optimization. It serves as a reference point for evaluating the performance improvements achieved by adding the proposed modules.
- PIR-XGBoost: This variant extends the baseline by integrating residual features derived from Physics-Informed Residual modeling. It is designed to evaluate whether the inclusion of physically consistent features enhances the accuracy and sensitivity of spoofing detection.
- PSO-XGBoost: In this configuration, the original feature set remains unchanged, while key hyperparameters of the XGBoost classifier are optimized using the Particle Swarm Optimization algorithm. This setup isolates the impact of intelligent hyperparameter tuning on model performance and generalization capability.
- PIR–PSO–XGBoost: This is the full proposed framework, which combines both physics-informed residual construction and swarm-based hyperparameter optimization. By enhancing feature representation and model calibration simultaneously, it aims to achieve optimal classification accuracy, robustness, and generalization in GPS spoofing detection tasks.

**Figure 5:**
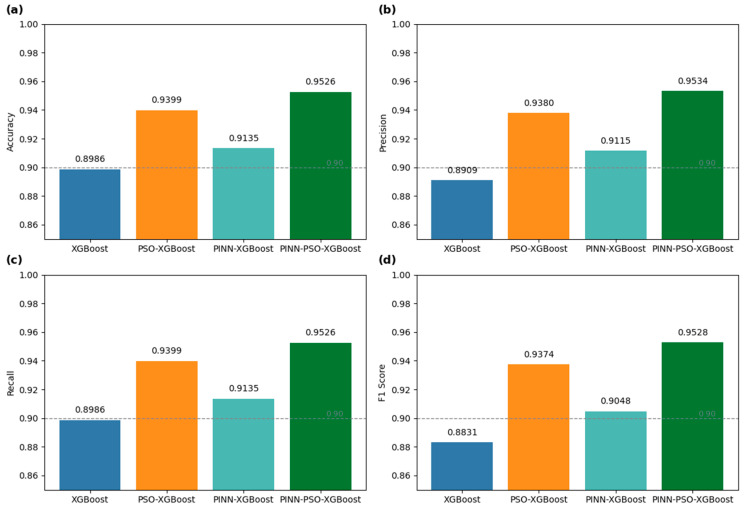
Accuracy, Precision, Recall and F1_Score of four models on the GPS signal spoofing dataset. (**a**) Accuracy results. (**b**) Precision results. (**c**) Recall results. (**d**) F1-score results.

All four model configurations were evaluated using the same dataset and consistent train–test partitioning to ensure fairness and comparability. Performance was assessed across multiple metrics to comprehensively reflect each model’s detection capability and overall stability in GPS spoofing scenarios. The comparative results for all configurations are presented in Figure 5.

As illustrated in Figure 5a, the baseline XGBoost model achieves an accuracy of 89.86%, reflecting its limited effectiveness in detecting spoofing-related patterns. When incorporating Physics-Informed Residual features, the accuracy increases to 91.35%, demonstrating the enhanced sensitivity of the model to abnormal signal behavior through physics-consistent feature representations. The application of hyperparameter optimization via Particle Swarm Optimization further boosts the accuracy to 93.99%, highlighting the role of intelligent optimization in improving the model’s generalization and discriminative power. The complete PIR–PSO–XGBoost framework achieves the highest accuracy of 95.26%, validating the combined advantages of physics-guided feature modeling and swarm-based hyperparameter tuning.

Figure 5b presents the precision results. The baseline model records a precision of 89.09%, which improves to 91.15% with PIR–XGBoost, thereby reducing false positives. PSO–XGBoost further increases precision to 93.80%, while the full PIR–PSO–XGBoost framework achieves the highest precision at 95.34%.

Figure 5c illustrates the recall performance, with values rising from 89.86% (baseline) to 91.35% (PIR–XGBoost), followed by 93.99% (PSO–XGBoost), and reaching 95.26% with the complete framework. This highlights the model’s ability to minimize missed detections effectively.

Figure 5d shows the trends in F1-score. The baseline model achieves an F1-score of 88.31%, while PIR–XGBoost and PSO–XGBoost achieve 90.48% and 93.74%, respectively. The fully integrated PIR–PSO–XGBoost model reaches the highest F1-score of 95.28%, indicating a superior balance between precision and recall.

Overall, Figure 5 comprehensively demonstrates the effectiveness and complementarity of the two core modules in the proposed framework. The physics-informed residual modeling module introduces domain knowledge, enhancing the model’s responsiveness to subtle spoofing anomalies. The PSO-based optimization module automates hyperparameter tuning, improving both adaptability and convergence. The synergy between these components enables the PIR–PSO–XGBoost framework to achieve state-of-the-art detection performance in terms of accuracy, stability, and robustness, underscoring its strong practical potential in complex GPS spoofing environments.

## 6. Discussion

The proposed PIR–PSO–XGBoost framework demonstrates superior performance in GPS spoofing detection. Experimental results show that it consistently outperforms traditional shallow learning models, such as Logistic Regression and K-Nearest Neighbors, as well as advanced deep learning architectures, including LSTM, CNN, and RNN, across multiple evaluation metrics, including accuracy, precision, recall, and F1-score. The framework achieves a classification accuracy of 95.26%, significantly outperforming the baseline XGBoost model (89.86%) and surpassing variants enhanced with either physics-informed residual features or particle swarm-based optimization alone. These results validate the synergistic advantage of integrating physics-guided feature engineering and intelligent hyperparameter tuning, enhancing detection robustness and reliability.

In terms of feature representation, incorporating Physics-Informed Residual features substantially improves the model’s sensitivity to physical anomalies. Rather than relying solely on raw GNSS inputs, the framework introduces physically grounded descriptors—such as carrier phase deviations, pseudorange inconsistencies, and fluctuations in signal-to-noise ratio—that allow the model to detect subtle perturbations and abnormal dynamics induced by spoofing attacks. At the same time, the integration of Particle Swarm Optimization automates the adjustment of key XGBoost hyperparameters (e.g., maximum depth, learning rate, number of estimators), eliminating the inefficiencies of manual tuning and improving adaptability to varying data conditions, ultimately enhancing generalization performance.

From an engineering perspective, the proposed framework offers strong flexibility, computational efficiency, and interpretability, making it suitable for deployment on platforms with constrained resources. By avoiding the need for deep neural network training, it reduces computational overhead while maintaining high accuracy. Moreover, the interpretability of residual features and the built-in feature importance analysis of the XGBoost-based classifier provide actionable insights for system diagnostics and operational maintenance.

Despite its high performance, several limitations remain. In real-world UAV applications, challenges such as data drift, non-stationary environments, and interference from multiple signal sources are common. These conditions necessitate evaluation in more dynamic and complex scenarios. Additionally, the current residual construction strategy relies on predefined physical equations and temporal statistics extracted via a sliding window mechanism. While effective, this approach limits the system’s ability to learn adaptive representations and select the most informative features. Future work could explore integrating neural network-based attention mechanisms to better capture temporal dependencies and context-aware patterns. Furthermore, although particle swarm optimization provides robust global search capabilities, it is prone to premature convergence in high-dimensional spaces. Alternative strategies, such as differential evolution or ant colony optimization, may offer improved convergence stability and efficiency. Collectively, these enhancements have the potential to further improve the practical applicability, scalability, and generalization capacity of the proposed framework for safeguarding GNSS-based navigation systems.

## 7. Conclusions

This study proposes a GPS spoofing detection framework, PIR–PSO–XGBoost, which integrates Physics-Informed Residual modeling, Particle Swarm Optimization, and XGBoost-based classification to achieve a balance between detection accuracy, interpretability, and computational efficiency. By constructing physically consistent residual features from GNSS observables and performing global hyperparameter tuning, the framework outperforms traditional shallow models and deep learning approaches, achieving a classification accuracy of 95.26% and an F1-score of 95.28% on a real-world UAV spoofing dataset. Compared to existing feature-engineered or black box models, PIR–PSO–XGBoost improves generalization across spoofing scenarios while maintaining a lightweight deployment capability for real-time applications. Future work will focus on extending the framework to address more complex threats, such as mobile or multi-antenna spoofing systems, and validating its real-time performance on embedded UAV platforms with hardware and communication constraints. Additional improvements may also involve integrating domain-specific signal processing knowledge and multi-sensor fusion to further enhance robustness in dynamic and adversarial environments.

## Figures and Tables

**Table 1:** Comparative summary of existing GPS spoofing detection methods and their limitations.

Study	Model Type	Physical Knowledge	Interpretability	Adaptability	Cost
Sun et al. [9]	Deep Learning	×	×	Medium	High
Talaei et al. [10]	Ensemble Learning	×	✓	Medium	Medium
Alanazi et al. [13]	Hybrid DNN	×	×	High	High
Al-Sabbagh et al. [15]	Genetic + DL	×	×	Medium	Medium
Abrar et al. [20]	Statistical Model	Partial	✓	Medium	Low
This study	Hybrid ML	✓ (explicit)	✓	High	Low

×: Not Applicable, ✓: Applicables.

**Table 2:** List of Features.

Feature Name	Feature Description
res_CP_PC	Absolute difference between the CP and PC; indicates signal inconsistency between phase and code observations, which may be disrupted during spoofing.
res_PIP_PQP	Absolute difference between PIP and PQP components; reflects channel level incoherence, which may emerge under spoofing induced signal distortion.
res_DO_PD	Absolute difference between DO and PD rate; captures inconsistencies in velocity estimation, often altered during spoofing attacks.
res_EC_LC	Absolute difference between EC and LC; highlights code tracking asymmetry, potentially caused by multipath or forged signal injection.
res_CN0_jump	Magnitude of abrupt change inC/N0 between consecutive epochs; designed to detect sudden fluctuations in signal strength, often induced by spoofed or jammed signals.
res_TOW	Temporal difference in TOW between successive observations; deviations may signal unsynchronized or manipulated time stamps.
res_TCD_jump	Absolute difference in TCD across adjacent epochs; captures abnormal changes in Doppler frequency tracking behavior.
res_RX_var	Rolling variance of Received Signal Strength over a fixed time window; higher variance suggests unstable reception, possibly linked to spoofing or interference.
res_CN0_slope	Difference in C/N0 between the current and a previous time window; serves as a temporal trend indicator of signal quality degradation under attack conditions.

**Table 3:** Range of PSO Hyperparameters.

Hyperparameter	Description	Range	Unit
Max Depth	Maximum depth of the trees used in XGBoost.	(3, 10)	N/A
Learning Rate	Step size at each iteration while moving toward a minimum.	(0.01, 0.3)	N/A
Subsample	Fraction of samples used for each boosting round.	(0.6, 1.0)	Percentage (%)
Colsample By Tree	Fraction of features used for each tree.	(0.6, 1.0)	Percentage (%)
Gamma	Minimum loss reduction required to make a further partition.	(0.0, 5.0)	N/A
Reg Lambda	L2 regularization term for boosting model.	(0.0, 5.0)	N/A
Number of Estimators	Number of boosting rounds.	(50, 200)	Rounds

**Table 4:** GPS Spoofing Detection Dataset Sample Number Distribution.

Samples	Output Label Value	Number of Instances
Normal	0	397,825
Simplistic Spoofing	1	36,458
Intermediate Spoofing	2	44,232
Sophisticated Spoofing	3	32,015
Total	510,530	

**Table 5:** List of UAV dataset parameters.

Feature	Description
Pseudo Random Noise (PRN)	Identifies each satellite uniquely to distinguish their signals.
Carrier Doppler (DO)	Used to infer satellite radial velocity from carrier frequency shift caused by relative motion.
Pseudo Range (PD)	Used to estimate the geometric distance between the satellite and receiver based on signal propagation time.
Receiver Time (RX)	Provides internal timing reference for aligning receiver time with GPS system time.
Time Of the Week (TOW)	Serves as a timestamp for synchronizing GPS data with system time.
Carrier Phase Cycle (CP)	Used for precise positioning and phase measurements by tracking accumulated carrier cycles.
Early Correlator (EC)	Used to fine tune signal tracking and improve timing accuracy by correlating early code.
Late Correlator (LC)	Assist in signal tracking and error correction by correlating late code components.
Prompt Correlator (PC)	Performs core code correlation for GPS signal synchronization.
Prompt Quadrature Component (PQP)	Used to improve detection accuracy as part of the signal’s quadrature component.
Tracking Carrier Doppler (TCD)	Defines effective Doppler range and corrects frequency shift during signal tracking.
Carrier to Noise Ratio (C/N0)	Used to evaluate GPS signal quality by comparing carrier signal power with noise power.

**Table 6:** Experimental results of ensemble models.

Model	Accuracy (%)	Precision (%)	Recall (%)	F1-Score (%)
LR	77.89	62.47	77.89	68.25
KNN	85.40	84.77	85.40	84.94
XGBoost	89.86	89.09	89.86	88.31
LSTM	89.99	89.96	89.99	89.90
MLP	91.49	91.94	91.49	91.51
CNN	86.46	85.55	86.46	85.42
RNN	89.28	88.91	89.28	89.01
PIR–PSO–XGBoost	95.26	95.34	95.26	95.28

## Data Availability

The original contributions presented in this study are included in the article. Further inquiries can be directed to the corresponding author.

## References

[1] Bisio I., Garibotto C., Haleem H., Lavagetto F., Sciarrone A. (2022). A Systematic Review of Drone Based Road Traffic Monitoring System. IEEE Access.

[2] Barmpounakis E., Geroliminis N. (2020). On the New Era of Urban Traffic Monitoring with Massive Drone Data: The *pNEUMA* Large-Scale Field Experiment. Transp. Res. Part C Emerg. Technol..

[3] Rabta B., Wankmüller C., Reiner G. (2018). A Drone Fleet Model for Last-Mile Distribution in Disaster Relief Operations. Int. J. Disaster Risk Reduct..

[4] Mohd Daud S.M.S., Mohd Yusof M.Y.P., Heo C.C., Khoo L.S., Chainchel Singh M.K., Mahmood M.S., Nawawi H. (2022). Applications of Drone in Disaster Management: A Scoping Review. Sci. Justice.

[5] Li Y., Liu C. (2019). Applications of Multirotor Drone Technologies in Construction Management. Int. J. Constr. Manag..

[6] Chen H., Hu Z., Solak S. (2021). Improved Delivery Policies for Future Drone-Based Delivery Systems. Eur. J. Oper. Res..

[7] Hartmann K., Steup C. The Vulnerability of UAVs to Cyber Attacks—An Approach to the Risk Assessment. Proceedings of the 2013 5th International Conference on Cyber Conflict (CYCON 2013).

[8] Feng Z., Guan N., Lv M., Liu W., Deng Q., Liu X., Yi W. (2020). Efficient Drone Hijacking Detection Using Two-Step GA-XGBoost. J. Syst. Archit..

[9] Sun Y., Yu M., Wang L., Li T., Dong M. (2023). A Deep-Learning-Based GPS Signal Spoofing Detection Method for Small UAVs. Drones.

[10] Talaei Khoei T., Ismail S., Kaabouch N. (2022). Dynamic Selection Techniques for Detecting GPS Spoofing Attacks on UAVs. Sensors.

[11] Wu S., Li Y., Wang Z., Tan Z., Pan Q. (2023). A Highly Interpretable Framework for Generic Low-Cost UAV Attack Detection. IEEE Sens. J..

[12] Alhoraibi L., Alghazzawi D., Alhebshi R. (2024). Detection of GPS Spoofing Attacks in UAVs Based on Adversarial Machine Learning Model. Sensors.

[13] Alanazi A. (2024). SSRL-UAVs: A Self-Supervised Deep Representation Learning Approach for GPS Spoofing Attack Detection in Small Unmanned Aerial Vehicles. Drones.

[14] Al-Syouf R., Aljarrah O.Y., Bani-Hani R., Alma’aitah A. (2025). Ensemble Machine Learning Models Utilizing a Hybrid Recursive Feature Elimination (RFE) Technique for Detecting GPS Spoofing Attacks Against Unmanned Aerial Vehicles. Sensors.

[15] Al-Sabbagh A., El-Bokhary A., El-Koussa S., Jaber A., Elkhodr M. (2025). Enhancing UAV Security Against GPS Spoofing Attacks Through a Genetic Algorithm-Driven Deep Learning Framework. Information.

[16] Xue N., Niu L., Hong X., Li Z., Hoffaeller L., Pöpper C. (2020). DeepSIM: GPS Spoofing Detection on UAVs Using Satellite Imagery Matching. Proceedings of the 36th Annual Computer Security Applications Conference.

[17] Davidovich B., Nassi B., Elovici Y. (2022). Towards the Detection of GPS Spoofing Attacks against Drones by Analyzing Camera’s Video Stream. Sensors.

[18] Basan E., Basan A., Nekrasov A., Fidge C., Sushkin N., Peskova O. (2022). GPS-Spoofing Attack Detection Technology for UAVs Based on Kullback–Leibler Divergence. Drones.

[19] Mouzai M., Riahla M.A., Keziou A., Fouchal H. (2025). Exploring Multi-Channel GPS Receivers for Detecting Spoofing Attacks on UAVs Using Machine Learning. Sensors.

[20] Hu J., Ammar M., Hussain B.Z., Kim J., Khan I. (2025). Reinforcement Learning Driven Integrated Detection and Mitigation of UAV GPS Spoofing Attacks. IEEE Internet Things J..

[21] Abrar M.M., Youssef A., Islam R., Satam S., Latibari B.S., Hariri S., Shao S., Salehi S., Satam P. (2024). GPS-IDS: An Anomaly-Based GPS Spoofing Attack Detection Framework for Autonomous Vehicles. arXiv.

[22] Song L.-K., Tao F., Li X.-Q., Yang L.-C., Wei Y.-P., Beer M. (2025). Physics-Embedding Multi-Response Regressor for Time-Variant System Reliability Assessment. Reliab. Eng. Syst. Saf..

[23] A DATASET for GPS Spoofing Detection on Autonomous Vehicles|IEEE DataPort. https://ieee-dataport.org/documents/dataset-gps-spoofing-detection-autonomous-vehicles.

